# Functional networks and dynamics in human seizure activity

**DOI:** 10.1186/1471-2202-12-S1-P32

**Published:** 2011-07-18

**Authors:** Mark A Kramer, Eric D Kolaczyk, Uri T Eden, Sydney S Cash

**Affiliations:** 1 Department of Mathematics and Statistics, Boston University, Boston, MA, 02215, USA; 2 Departments of Neurology, Massachusetts General Hospital, Boston, MA, 02114, USA; 3 Harvard Medical School, Boston, MA, 02115, USA

## 

Epilepsy, the condition of recurrent unprovoked seizures, is the world's most prominent serious brain disorder, affecting some 50 million people worldwide. For an estimated 30% of these patients, seizures remain poorly controlled despite maximal medical management. Moreover, control of epilepsy through medication and surgery often results in significant, sometimes debilitating, side effects. Advancing the therapeutic management of epilepsy requires a detailed understanding of the focal initiation and subsequent spread of seizure dynamics over a network of interconnected brain regions.

The human brain is naturally conceived as a network generating rhythmic fluctuations of coordinated neuronal activity. This “brain network” consists of two fundamental components: nodes (e.g., cortical columns) and edges (e.g., synaptic connections) that connect node pairs. In neuroscience, brain networks are typically divided into two categories: persistent structural networks based on anatomical connections between brain regions, and transient functional networks representing the coupling between dynamic activity recorded from separate brain areas. Ongoing research suggests common topological properties emerge in these brain networks, including small-worldness, hierarchical organization, and the presence of densely connected hubs [1]. In epilepsy --- perhaps best characterized as a disease of brain rhythms [2] --- the relationship between dynamic functional networks and pathological brain rhythms remains incompletely understood.

Here we present a general paradigm for the inference of dynamic functional networks from time series data, and apply this paradigm to the analysis of multielectrode invasive voltage recordings made directly from cortex and deep brain regions of human subjects during seizure. We first describe the implementation of this paradigm for a computationally efficient choice of coupling measure and consider the robustness of this measure for simulated data sets. We then apply this procedure to the analysis of electrocorticogram (ECoG) data recorded from a population of human subjects during seizure. We investigate how the dynamic functional networks evolve during seizure, and propose three new insights [3]: 1) At the spatial scale of ECoG recordings, brain regions decouple during seizure, 2) Network topologies coalesce at seizure onset and just before termination, while fragmenting during seizure, and 3) Similar functional network topologies appear from seizure-to-seizure.

We conclude that the seizure consists of characteristic neuronal dynamics (i.e., the rhythmic voltage fluctuations of seizure) and network dynamics. Our ongoing work focuses on understanding the relationships between the dynamic networks and rhythms of the seizure, specifically how changing network connectivity (i.e., dynamics of networks) affects neuronal activity produced at individual nodes (i.e., dynamics on networks). Combined, a deeper understanding of the network dynamics and neuronal rhythms of the seizure promises to provide new insights, and perhaps therapeutic strategies, for the treatment of epilepsy.
